# A Web-Based, Social Networking Beginners’ Running Intervention for Adults Aged 18 to 50 Years Delivered via a Facebook Group: Randomized Controlled Trial

**DOI:** 10.2196/jmir.7862

**Published:** 2018-02-26

**Authors:** Jemma Looyestyn, Jocelyn Kernot, Kobie Boshoff, Carol Maher

**Affiliations:** ^1^ School of Health Sciences University of South Australia Adelaide Australia; ^2^ Alliance for Research in Exercise, Nutrition, and Physical Activity University of South Australia Adelaide Australia

**Keywords:** social network, health behavior, program evaluation, Internet, physical activity

## Abstract

**Background:**

Online social networks continue to grow in popularity, with 1.7 billion users worldwide accessing Facebook each month. The use of social networking sites such as Facebook for the delivery of health behavior programs is relatively new.

**Objective:**

The primary aim of this study was to determine the effectiveness of a Web-based beginners’ running program for adults aged 18 to 50 years, delivered via a Facebook group, in increasing physical activity (PA) and cardiorespiratory fitness.

**Methods:**

A total of 89 adults with a mean age of 35.2 years (SD 10.9) were recruited online and via print media. Participants were randomly allocated to receive the UniSA Run Free program, an 8-week Web-based beginners’ running intervention, delivered via a closed Facebook group (n=41) that included daily interactive posts (information with links, motivational quotes, opinion polls, or questions) and details of the running sessions; or to the control group who received a hard copy of the running program (n=48). Assessments were completed online at baseline, 2 months, and 5 months. The primary outcome measures were self-reported weekly moderate to vigorous physical activity (MVPA) and objectively measured cardiorespiratory fitness. Secondary outcomes were social support, exercise attitudes, and self-efficacy. Analyses were undertaken using random effects mixed modeling. Compliance with the running program and engagement with the Facebook group were analyzed descriptively.

**Results:**

Both groups significantly increased MVPA across the study period (*P*=.004); however, this was significantly higher in the Facebook group (*P*=.04). The Facebook group increased their MVPA from baseline by 140 min/week versus 91 min for the control at 2 months. MVPA remained elevated for the Facebook group (from baseline) by 129 min/week versus a 50 min/week decrease for the control at 5 months. Both groups had significant increases in social support scores at 2 months (*P*=.02); however, there were no group by time differences (*P*=.16). There were no significant changes in the other outcomes. A process evaluation revealed relatively high levels of engagement with the Facebook group during the 8-week intervention (eg, mean number of interactions 35 [SD 41]).

**Conclusions:**

An 8-week beginners’ running program delivered through Facebook produced sizable and sustained changes in weekly MVPA and received strong engagement and positive feedback from participants. Future research investigating this intervention approach is warranted in other populations and health behaviors.

**Trial Registration:**

Australian New Zealand Clinical Trials Registry ACTRN12616001500448; https://www.anzctr.org.au/Trial/Registration/TrialReview.aspx?id=371607&isReview=true (Archived by WebCite at http://www.webcitation.org/6xSAuz4NW)

## Introduction

Physical inactivity is among the leading risk factors for mortality and has been linked to an increased risk of chronic diseases such as type 2 diabetes, cardiovascular disease, and certain cancers including breast and colon cancer [1]. Worldwide, physical inactivity is estimated to cost global health care systems USD 53.8 billion dollars [2].

The Australian Government Department of Health [3] recommends that adults aged between 18 and 64 years engage in 150 to 300 weekly minutes of moderate physical activity (PA; eg, brisk walking), or 75 to 150 weekly minutes of vigorous PA (eg, jogging or singles tennis), or the equivalent combination of moderate to vigorous physical activity (MVPA).

Jogging or running affords many practical benefits: it is inexpensive, it requires little to no equipment, it is time efficient, and can easily be incorporated into daily routine [4]. Regular running has been linked to positive physical and psychological outcomes such as improving cardiovascular fitness, maintaining or improving blood pressure, and preventing or managing mental illnesses such as depression [5]. In addition, vigorous PA (eg, running or jogging) is reported to have greater cardioprotective benefits than moderate activity (eg, walking) [6].

Previous research has explored running as a means of increasing physical and emotional well-being [7,8]. The delivery of jogging interventions vary, with some programs using one or a combination of face-to-face, group-based, Internet- or print-based delivery methods [7,8]. Online social networking is a unique delivery method that has yet to be explored for jogging interventions.

Online social networks such as Facebook are incredibly popular, accounting for a quarter of all time spent online [9,10]. Globally, Facebook is the most popular online social networking site, with 2 billion active users each month and 15 million monthly Australian users [11]. Facebook’s popularity and its ability to impart social influence offers promise for the delivery of low-cost, mass-scale health behavior interventions.

Two systematic reviews examining the use of online social networks in behavior change interventions found modest evidence of effectiveness [12,13]. Many studies to date have used Facebook as a component of a more complex intervention and offered a range of additional intervention materials and resources [13], for example, an online discussion page [14], a self-monitoring website [15], pedometers [16-18], accelerometers [19], or cook books [20]. This makes it difficult to disentangle the effectiveness of the online social networking component from other intervention components.

Studies specifically looking at the use of online social networks for PA interventions have reported mixed results. Facebook apps have shown promising results for the delivery of health interventions in terms of efficacy and engagement [17,21] but require considerable expertise and funding to develop.

Cavallo and Valle [15,18] explored the use of Facebook groups, a pre-existing Facebook feature, for PA intervention delivery. In both studies, the Facebook group was a component of the intervention, with participants also having access to a separate online website. Comparison groups received alternative interventions (website only [15] and Facebook self-help group [18]). The primary outcomes were social support for PA [15] and self-reported PA [15,18]—no significant groups by time differences were reported.

To the best of our knowledge, no studies have explored the use of a Facebook group as the sole means of delivering a PA intervention. Given Facebook’s popularity and the simplicity and cost-effectiveness of using pre-existing Facebook features such as Facebook groups, further research is required to evaluate the effectiveness of this intervention delivery method.

The primary objective of this study was to determine the effectiveness of an 8-week beginners’ running program (*UniSA Run Free*) delivered entirely via a Facebook group in increasing PA and cardiorespiratory fitness in adults aged 18 to 50 years. The secondary objectives were as follows: (1) to determine the effectiveness of this program in improving PA attitudes, self-efficacy, and social support; (2) to determine engagement and feasibility of the program; and (3) to examine whether changes in MVPA are related to demographics, baseline characteristics, and engagement.

## Methods

### Overview

This parallel randomized controlled trial (RCT), allocation ratio 1:1, was approved by the University of South Australia Human Research Ethics Committee (protocol number: 0000033766). Data collection took place in Australia from January 2016 to August 2016. Data analysis occurred from August 2016 to November 2016. Participants provided informed consent online before commencing the study. The study was designed and is reported following the Consolidated Standards of Reporting Trials guidelines [22] and is registered with the Australian and New Zealand Clinical Trials Registry, protocol number: ACTRN12616001500448.

### Recruitment and Randomization

Participants were recruited through a variety of advertising methods including online (via Facebook advertising) and via print media. Participants were eligible to take part in the study if they met the following criteria: (1) aged between 18 and 50 years, (2) Australian residents, (3) current Facebook users, (4) able to read and understand English, and (5) not participating in a regular running program. Individuals were excluded if they had a medical condition that would prevent them from participating in a running program and if they were pregnant or planning to become pregnant within the next 5 months.

Interested participants were directed to the UniSA Run Free Facebook page that provided study information and invited potential participants to register their interest by privately texting their contact details. An online survey was used to confirm eligibility (participants were asked questions related to the eligibility criteria), collect written informed consent, and perform baseline assessments. Participants were formally enrolled once they completed baseline surveys. Upon enrollment, they were randomly allocated (by the primary researcher, JL) to the intervention (UniSA Run Free program) or control condition (self-directed running program) using a computer-generated random number sequence with allocation concealment (opaque envelopes were used for allocation concealment).

Details regarding the honorarium for this study were provided in the participant information sheet and consent form. All participants who enrolled in the study and who completed all three assessments were placed in a prize draw for an Aus $200 gift voucher.

### Interventions

#### UniSA Run Free (Intervention Condition)

UniSA Run Free is an 8-week beginners’ running program delivered via a closed Facebook group. This program is based on social cognitive theory (SCT), which encompasses key constructs underpinning the UniSA Run Free Facebook group content and the outcome measures selected for this study [23,24].

SCT emphasizes the interaction of three factors that may affect or be affected by each other, referred to as *reciprocal determinism* [23,24]. These include:

1. Environmental factors—a Facebook group was chosen for the intervention delivery as it provides a social environment to promote peer encouragement, sharing, and support.
2. Personal factors—this program was targeted at *beginner runners* (people with similar skill levels; those participating in a regular running program were excluded).
3. Behavioral factors—the program was graded to allow for incremental gains in running skills and fitness (behavioral change).

**Figure 1:**
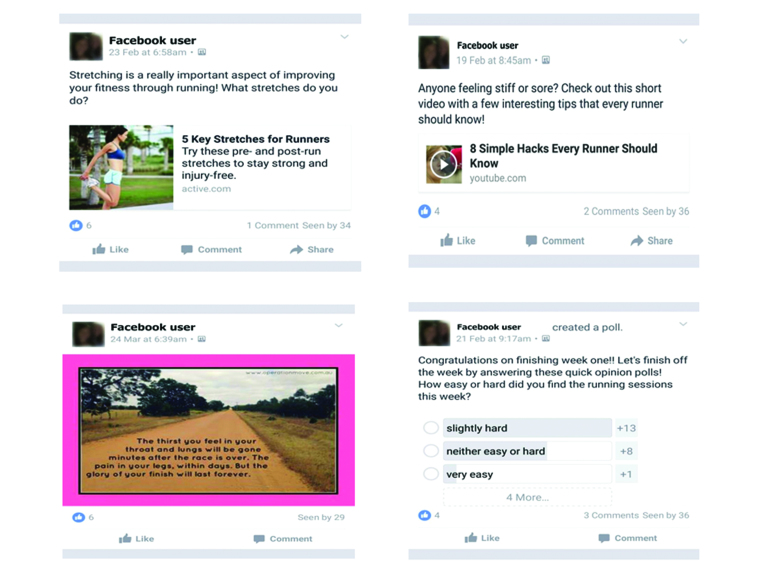
Examples of the UniSA Run Free Facebook group posts.

An additional key construct of SCT is self-efficacy, which refers to a persons’ self-confidence to carry out the behavior [23,24]. Self-efficacy was promoted through short-term (session goals) and long-term goals (running 30 consecutive minutes by the end of the program) and Facebook posts (see Figure 1) offering information and motivational material.

**Figure 2:**
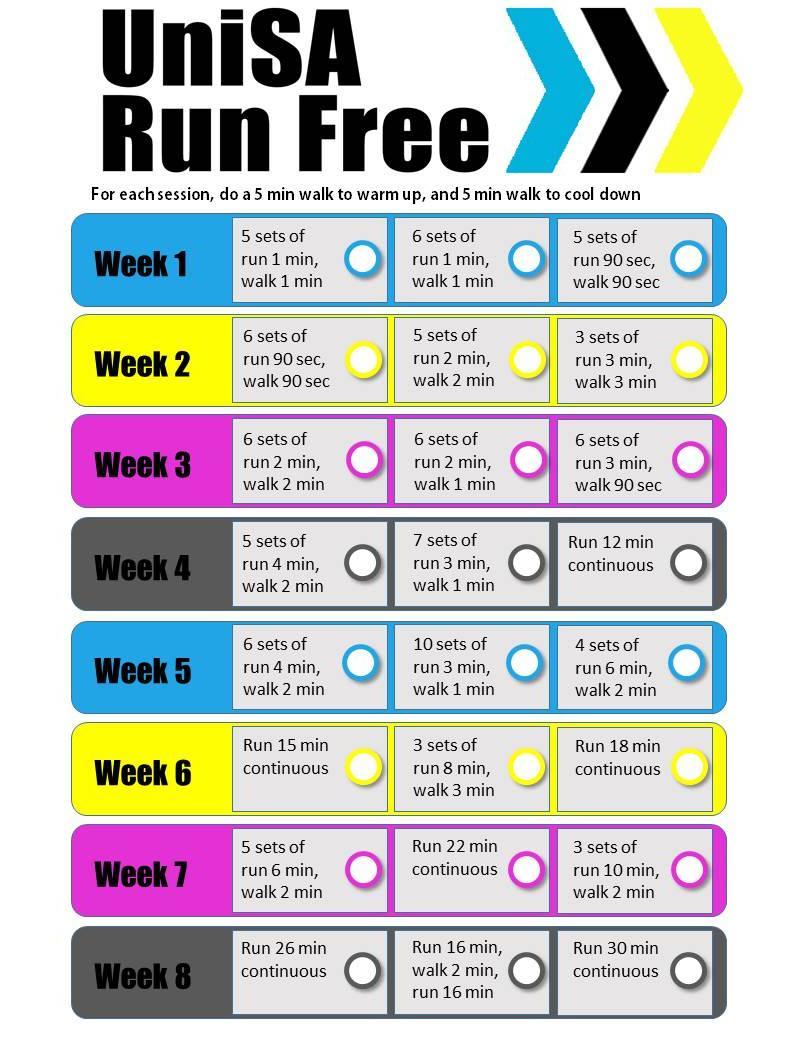
Running program A4 size fridge magnet.

The running program consisted of three interval training sessions per week; each session included a warm up, main activity, and cool down (see Figure 2). The program was created by health professionals at the University of South Australia (CM and JK) in collaboration with fitness experts, to ensure it progressed in a safe and achievable manner for novice runners. The end goal of the program was for the participants to run continuously for 30 min. The UniSA Run Free program was delivered entirely via a closed Facebook group (only participants randomized to the intervention group could access this). The running sessions were posted onto the Facebook group weekly. In addition, participants were posted an A4 fridge magnet (see Figure 2) outlining the running program in its entirety so that they could tick sessions off as they were completed.

Each day (for the duration of the 8-week program), the group facilitator (JL) posted a message to the closed Facebook group. These posts were informative and encouraged social interaction including asking participants to post photos, providing information with links, motivational quotes, opinion polls, and posts prompting participants to answer questions and interact with others (see Figure 1).The type of post and the content was varied to maximize participants’ engagement and interest. Participants were encouraged to interact with the facilitator’s posts and contribute their own posts to the group. The facilitator ensured that her responses to participants’ posts were consistent (ie, liking posts).

#### Self-Directed Running Program (Control Condition)

Participants randomized to the control condition were given a self-directed running program only to follow (individually) and did not have access to the Facebook group. The running program, which was provided in its entirety, was posted to participants in the form of an A4 sized fridge magnet (see Figure 2) and included the same running program structure as for the UniSA Run Free group, with participants encouraged to tick off sessions as they were completed.

Participants in the intervention and control conditions commenced the running program in February 2016.

### Experiment Procedure

There were three assessment time points: baseline, 2 months (coinciding with the last week of the running program), and 5 months (3 months post program). All assessments were self-administered and completed remotely (online). Blinding of participants was not possible because of the nature of the intervention. Blinding of assessors was not applicable, as assessments were self-administered.

#### Outcome Measures

The primary outcomes were self-reported MVPA and cardiorespiratory fitness, and secondary outcomes were self-efficacy, exercise attitudes, and social support. A process evaluation was also undertaken to investigate engagement and feasibility of the UniSA Run Free Facebook program.

#### Self-Reported Moderate to Vigorous Physical Activity

Self-reported total weekly MVPA was measured via the Active Australia Survey (AAS) [25]. The AAS has been widely used and validated with an Australian population and is comprised of eight questions that measure the frequency and amount of time spent in MVPA within the past 7 days [25]. As per AAS protocol, MVPA was determined by calculating walking time + other moderate activity time + 2 x vigorous activity, with each individual item truncated at a maximum of 840 min per week and total PA truncated at a maximum of 1680 min, to reduce over-reporting [25]. The AAS has been shown to have moderate reliability (rho=.56-.64) and moderate validity compared with pedometry and accelerometry (rho=.43 and rho=.52, respectively) [25,26].

#### Cardiorespiratory Fitness

Cardiorespiratory fitness was measured via the YMCA step test [27]. Laboratory-based testing is considered to be the gold standard for measuring cardiorespiratory fitness; however, this requires sophisticated equipment, is time-consuming, and expensive. Submaximal tests such as the YMCA step test have been designed to be self-administered in free-living conditions (as per this study) and have been found to be a valid (*r*=.61, compared with laboratory VO_2_ max testing [25]) means of estimating cardiorespiratory fitness [28].

This YMCA step test requires participants to step up and down continuously on a 30-cm step for 3 min and at completion, measure their heart rate (total beats in a 60-sec period). To ensure that participants followed the correct procedure, a YouTube clip guiding participants through the YMCA step test was specifically created for the purpose of this study [29], and participants were provided with instructions for measuring radial artery heart rate.

#### Secondary Outcomes

SCT was used to guide the intervention design; therefore, the secondary outcomes were selected to measure these constructs (self-efficacy, attitudes, and social support). The Self-Efficacy Barriers to Exercise Measure comprises of 13 statements asking participants to rank how confident they felt in continuing to exercise when certain issues occurred [30]. The internal consistency of this measure is 0.93 [30]. As per standard procedure, the Self-Efficacy Barriers to Exercise Measure was scored by adding the ratings for each response and dividing the sum by 13 [31].

The Exercise Attitude Questionnaire-18 consists of 18 statements, where participants rank their attitude to exercise on a 5-point Likert scale [32]. Internal consistency of this instrument is 0.74 and test-retest reliability intraclass correlation coefficient=0.90 [32]. As per guidelines, all negative statement scores were reversed and all results calculated based on the mean score, giving a score ranging from 0 to 100 [33].

The social support and exercise survey was used to evaluate the amount of social support participants received in regards to their PA [34]. It includes 13 questions where participants rank their experiences on a 5-point Likert scale (none to very often). Internal consistency of the combined family and friends score is 0.79 [34]. The scale is scored by calculating the sum of all items [34].

The following baseline demographic characteristics were also collected: age (years), weight (kg), height (cm), and highest level of education (high school, technical and further education certificate or diploma, or a university degree or higher).The self-reported height and weight information was used to calculate participants’ body mass index (BMI). BMI was categorized into the following: underweight (<18.5 kg/m^2^), normal (18.5 to <25.0 kg/m^2^), overweight (25.0 to <30.0 kg/m^2^), and obese (≥30.0 kg/m^2^) [35].

#### Process Evaluation

Process evaluation occurred concurrently with the RCT and assessed engagement with and feasibility of the UniSA Run Free program. Participants’ compliance with the running program was determined by participants indicating in the 2-month survey the number of running sessions completed. From this, the percentage of completed running sessions was calculated. Intervention participants’ engagement with the Facebook group was measured in two ways: (1) the Facebook group page was audited to determine the number of interactions (posts, comments, likes, poll votes, and photos uploaded) per participant and (2) in the 2-month survey, participants were asked to self-report the number of visits to the group page (to capture occasions where the page was viewed without interactions). Additionally, the feedback survey contained seven items regarding the perceived usefulness, relevance, and motivation benefit of the Facebook group.

### Statistical Analysis

Participants’ baseline characteristics were analyzed descriptively. Changes in primary and secondary outcomes from baseline to the 2- and 5-month assessments were analyzed using random effects mixed modeling. Analyses were conducted using generalized linear mixed models (GLMMs) in Statistical Package for the Social Sciences (SPSS) version 21 (IBM Corp), with the individual entered as a random effect and group, time, and group x time interaction entered as fixed effects. Data analysis was completed on an intention-to-treat basis, where all participants randomized at the commencement of the trial were retained for analysis regardless of compliance [36]. The GLMM function is able to handle missing data; therefore data imputation was not needed. Effect size differences between groups at 2 and 5 months were calculated using Cohen *d* [37].

Compliance and engagement with the UniSA Run Free program were described descriptively. A subgroup analysis using GLMM was undertaken within the UniSA Run Free group to determine if change in MVPA was related to key sociodemographic characteristics (age and sex), baseline characteristics (BMI, fitness, and MVPA), as well as compliance with the running program and engagement with the Facebook group (ie, liking or commenting on the facilitators posts). For these analyses, the predictor variables were dichotomized into high and low categories based on the median splits. Specifically, compliance was categorized into high (≥70% of running sessions completed) and low (<70%), fitness categorized into high (<100 beats per minute, BPM) and low (≥100 BPM), engagement into high (≥15 interactions) and low (<15 interactions), BMI into high (≥25 kg/m^2^) and low (<25 kg/m^2^), and age into older (≥35 years) and younger (<35 years). Baseline PA was dichotomized on the basis of meeting the PA guidelines (≥150 weekly minutes) and failing to meet guidelines (<150 weekly minutes).

A priori sample size calculations suggested that a sample of 114 participants would be sufficient to detect a moderate effect size (*d*=0.4), assuming a two-group design with three repeated measures, 80% power, and an alpha of .05. Because the study was conducted in the context of a student research project, there were time constraints on participant recruitment. A total of 89 participants were recruited with post-hoc power analyses, suggesting that this sample had 64% power to detect effect size differences of *d*=0.4.

## Results

### Participants

A total of 210 potential participants registered their interest in the study; however, only 89 met the participant criteria and completed baseline assessment and were therefore formally enrolled. Of these 89 participants, 41 (46%) were randomized to the intervention group and 48 (53%) to the control group (based on the computer generated number sequence). Three-quarters (78% [69/89]) completed the 2-month assessments, whereas two-thirds (65% [58/89]) completed the 5-month assessments.

**Figure 3:**
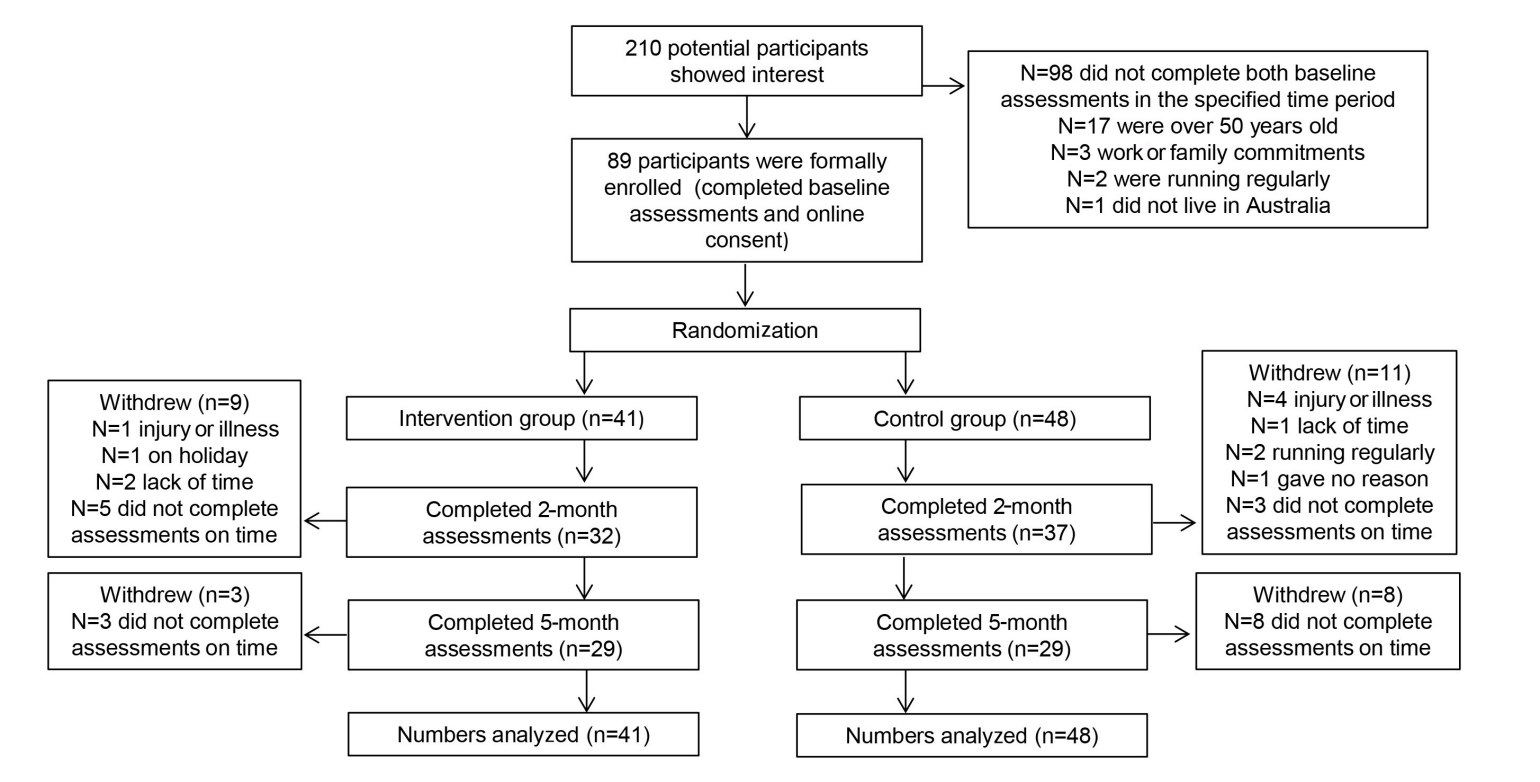
Participant flow through the study.

Twelve participants formally withdrew from the study for various reasons, as listed in Figure 3.

Participants’ demographic and baseline characteristics are given in Table 1. Of the 89 participants, 71 (80%) were female, and the mean age was 35.2 years (SD 10.9). Overall, 37 participants (42% [37/89]) were within the normal BMI range [35], 28 were overweight (32% [28/89]), and 23 were obese (26 [23/89]). A total of 65 participants (73% [65/89]) were currently undertaking or had completed a university degree or higher. Participants reported getting a mean of 318 min (SD 278) of MVPA per week.

### Primary and Secondary Outcomes

The results for the primary and secondary outcome measures are shown in Table 2.

#### Self-Reported Moderate to Vigorous Physical Activity

There was a significant increase over time in MVPA in both the intervention and control groups (time effect *P*=.004). However, the increase was considerably larger in the intervention group (group x time effect of *P*=.04). From baseline to 2 months, the UniSA Run Free group increased their weekly MVPA by a mean of 140 min per week (SE 50 min), whereas the control group increased by 91 min (SE 47 min), equating to a between group effect size difference of *d*=1.01 in favor of the intervention group. At 5 months, the intervention groups’ MVPA remained elevated by a mean of 129 min per week (SE 49 min) compared with baseline, whereas the control groups’ MVPA fell to 50 min (SE 49 min) below baseline values. This equated to a between group effect size difference of *d*=3.65.

#### Cardiorespiratory Fitness

There was a nonsignificant trend for both groups to improve their cardiorespiratory fitness, denoted by a suggested decrease in mean BPM over time (time effect *P*=.12). However, there were no group by time differences (*P*=.76).

**Table 1:** Descriptive characteristics of the study sample at baseline (n=89). Arrows (↑ or ↓) indicate the desirable direction for each of the outcome measures.

Baseline characteristics		Control (n=48)	Intervention (n=41)
Age in years, mean (SD)		35.1 (10.9)	35.3 (11.2)
**Gender, n (%)**			
	Male	14 (29)	4 (10)
	Female	34 (71)	37 (90)
**Highest level of Education, n (%)**			
	High school	6 (13)	2 (5)
	Diploma or technical and further education certificate	13 (27)	3 (7)
	University degree or higher	29 (60)	36 (88)
Fitness, mean (SD) ↓		103 (26)	105 (27)
BMI^a^, mean (SD) ↓		26.7 (4.5)	27.6 (5.6)
Self-reported MVPA^b^(min/week), mean (SD) ↑		360 (301)	269 (242)
Self-efficacy^c^, mean (SD) ↑		42.6 (21.2)	44.9 (23.6)
Exercise attitude, mean (SD) ↑		62.5 (12.2)	62.99 (10.3)
Social support^d^, mean (SD) ↑		25.4 (9.7)	23.07 (8.8)

^a^BMI: body mass index.

^b^MVPA: moderate-to-vigorous physical activity.

^c^Self-Efficacy of Barriers to Exercise Measure.

^d^Social support and exercise survey.

**Table 2:** Outcome measures at baseline, 2-month follow-up, and 5-month follow-up. Arrows (↑ or ↓) indicate the desirable direction for each of the outcome measures.

Outcome measures		Assessment period, mean (SE)			Treatment effect, effect size (95% CI)			Group-by-time interaction, *F* (*P*)	
		Baseline	2 months	5 months		Baseline compared with 2 months	Baseline compared with 5 months		
**Self-reported MVPA^a^(min/week)** ↑									
	Intervention	269.0 (47.5)	409.5 (52.0)	398.3 (52.8)		1.01 (.56-1.45)	3.65 (2.94-4.30)		3.39 (.04^b^)
	Control	359.6 (43.9)	450.8 (48.3)	309.8 (52.1)					
**Fitness (BPM^c^)** ↓									
	Intervention	105.1 (4.2)	100.4 (4.6)	99.6 (4.7)		.08 (−.34 to .50)	−.08 (−.50 to .33)		.27 (.76)
	Control	103.2 (3.9)	96.4 (4.3)	100.2 (4.7)					
**Self-efficacy^d^** ↑									
	Intervention	44.9 (3.3)	41.6 (3.7)	44.6 (3.9)		−.20 (−.62 to .22)	.00 (−.42 to .42)		.56 (.58)
	Control	42.6 (3.1)	44.3 (3.4)	42.2 (3.8)					
**Exercise attitude^e^** ↑									
	Intervention	63.0 (1.7)	64.4 (1.8)	63.4 (1.9)		−.05 (−.47 to .36)	−.14 (−.56 to .28)		.24 (.79)
	Control	62.5 (1.5)	64.4 (1.6)	64.6 (1.8)					
**Social support^f^** ↑									
	Intervention	23.1 (1.5)	27.4 (1.6)	24.0 (1.6)		.41 (−.01 to .83)	.15 (−.27 to .57)		1.87 (.16)
	Control	25.4 (1.4)	26.1 (1.5)	24.9 (1.6)					

^a^MVPA: moderate-to-vigorous physical activity.

^b^Indicates statistical significance (*P*<.5).

^c^BPM: beats per minute.

^d^Self-Efficacy of Barriers to Exercise Measure.

^e^Exercise attitude questionnaire.

^f^Social support and exercise survey.

#### Secondary Outcomes

There were no group by time differences for any of the secondary outcomes (self-efficacy, exercise attitudes, or social support). Both groups significantly improved their social support across the intervention period (time effect *P*=.02), which appeared slightly larger in the intervention group; however, this was not statistically significant (group x time *P*=.16).

#### Process Evaluation

The process evaluation was completed for n=41 intervention participants and n=48 control participants. The mean number of running sessions reported as completed by the intervention participants was 17.3 (72% [SD 7.2]), whereas the corresponding number for the control group was 14.4 (60% [SD 8.1]) out of a maximum possible 24 sessions.

Six (19%) intervention participants reported visiting the Facebook group at least daily. All remaining participants reported visiting the group between one and six times per week (n=34; 78%), whereas one participant (3%) reported never visiting it.

Engagement with the Facebook group was measured by the total number of interactions per participant in response to posts made by the UniSA Run Free facilitator and those made by other participants over the 8-week program. The mean total number of interactions with the Facebook group was 34.7 (SD 40.7; median 15 [interquartile range 62.3]; range 0-158).This engagement data was positively skewed as eleven out of 41 participants had more than 50 interactions with the Facebook group.

During the 2-month survey, participants in the intervention group were asked to provide feedback on the UniSA Run Free program. Feedback was generally positive, with 63 per cent agreeing that the Facebook group helped them complete the running program. The feedback received about the posts made by UniSA Run Free facilitator was positive, with 75 per cent agreeing that the posts were supportive, and most participants agreed that the posts were relevant (66%) and motivating (66%). In relation to participant-initiated posts, most agreed that the posts were supportive (69%), relevant (59%), and motivating (59%).

### Subgroup Analysis

Subgroup analyses was undertaken to determine whether, within the intervention group, changes in MVPA were related to age, sex, highest level of education, percentage of running sessions completed, baseline BMI or fitness, engagement with the Facebook group, and baseline PA. Results showed that participants with high overall program compliance increased their MVPA significantly more than the participants with a low compliance (<70%; *P*=.03). In addition, participants who failed to meet PA guidelines at baseline (<150 weekly minutes) increased their MVPA significantly more (*P*=.04). Changes in MVPA appeared unrelated to age (*P*=.90), sex (*P*=.07), education (*P*=.95), baseline BMI (*P*=.89), and baseline cardiorespiratory fitness (*P*=.94).

### Adverse Effects

Throughout the 8-week intervention, 5 participants reported adverse events in the form of musculoskeletal lower limb injuries (intervention group n=2 and control group n=3). Three of these participants reported an exacerbation of a pre-existing condition, one participant sustained a knee injury from participating in another activity (nonstudy related), and the remaining participant sustained a new knee injury during a running session. All adverse effects were reported to the University of South Australia Human Ethics Committee.

## Discussion

### Principal Findings

The key findings of this study was that a beginners’ running program delivered via a Facebook group produced sizable and sustained changes in weekly MVPA compared with the same running program delivered in a self-administered format. Both groups reported a significant improvement in social support for PA, and there was a trend for both groups to improve their cardiorespiratory fitness, though this did not reach statistical significance. The running program delivered via Facebook achieved strong engagement, high compliance, and favorable feedback from participants.

Although some previous studies utilizing Facebook groups to deliver PA interventions have reported significant improvements in MVPA over time, to the best of our knowledge, no significant group by time effects have been noted [15,18]. The positive group by time effects for MVPA for this study may be related to a number of factors. First, the Facebook group was used to deliver all of the intervention materials; rather than being a component of a more complex intervention. Second, facilitator posts that varied in content and style were provided daily to encourage participant engagement. Third, the type of posts made, that is, posts containing questions, photos, and humor were guided by previous research, suggesting these post types are associated with higher engagement [38,39].

There was a nonsignificant trend for both groups to improve their fitness across the study period (time effect *P*=.12). Given that the study was underpowered and at risk of type 2 errors, the trend may in fact represent true improvement in fitness. Conversely, it is possible that an 8-week program was not long enough to see significant changes in this outcome. Wenger and Bell [40] suggest that it takes 10 to 11 weeks to improve cardiorespiratory fitness. The YMCA step test was selected, as it is easy for participants to self-administer, relatively safe (being a submaximal test), and can be completed with minimal special equipment. However, it is acknowledged that this is less reliable than laboratory-based cardiorespiratory fitness tests [41].

Relative to other online social networking interventions, participant engagement in UniSA Run Free was high; as indicated by a mean number of 35 (SD 41) total Facebook interactions, 19% of participants visiting the Facebook group daily, and 78% visiting between 1 and 6 times per week. In comparison, Wójcicki and colleagues [42] who investigated the feasibility of an 8-week Facebook group–delivered PA intervention in adolescents, reported low levels of group engagement, with only 27% of participants interacting with the Facebook group. Similarly, Napolitano and colleagues [20] reported only 24% of participants interacting with the Facebook group during an 8-week weight loss intervention. These differences in engagement may be because of the Facebook group being implemented differently in this study. Most other studies have used a Facebook group as a supplement to other intervention materials. In contrast, in our study, the Facebook group was central to the intervention and included all key intervention materials.

Retention rates for this study were also reasonably high at 78% at 2 months and 65% at 5 months. Similarly, high retention rates have been noted in previous Facebook-delivered PA interventions, ranging from 77% [43] to 96% [20]. The high retention rates for this delivery method may be related to its *real life* design, whereby participants can complete the intervention and assessments at home with minimal contact from research personnel, making it less intrusive and easier to fit around daily routines.

### Strengths and Limitations

This study had a number of methodological strengths. First, it was a parallel RCT, including intention-to-treat analysis, which is the gold standard in clinical trial design. Second, the comparison group were provided with an alternative intervention (hard copy of the running program), which allowed comparison of the two intervention delivery methods (hard copy vs Facebook group delivery) [44].

High levels of engagement and retention were also strengths and provide evidence of the feasibility of a Facebook group for intervention delivery. Furthermore, this delivery method enabled the intervention to be available to participants living all across Australia, thus, further demonstrating that online social networks have minimal geographic boundaries. Finally, results of the subgroup analyses found that the intervention was more effective in participants who failed to meet the minimum PA guidelines at baseline (150 min per week), indicating that this intervention was successful in assisting those most at risk of physical inactivity.

Key limitations of this study should also be addressed. Due to time restrictions with recruitment, this study was underpowered. Future work is needed to determine the effectiveness of this intervention with a larger sample, particularly for cardiovascular fitness and social support, which showed trends for improvement. Higher baseline MVPA of the control group (90.6 min/week higher than the intervention group) must also be acknowledged because of potential ceiling effect.

For practicality, all outcome measures were self-reported or self-administered. Self-reported measures are typically more prone to social desirability bias [45], and the self-administered nature of the step test reduces the ability to standardize test conditions and accuracy of heart rate measurement. In addition, determining participants’ social networking use (ie, frequency) at baseline would be beneficial as this may influence engagement and health behavior outcomes.

Finally, the somewhat homogenous nature of this sample (female and highly educated), which is typical of volunteer research studies, is also acknowledged [46,47]. This, along with the high baseline MVPA levels (for both groups), warrants caution in generalizing results. Further research is required to investigate the effectiveness of this intervention with other population subgroups (eg, teenagers and individuals over 50 years).

### Conclusions

Previous research has found modest evidence supporting Facebook groups as a delivery method for PA interventions. Many previous studies have offered additional intervention materials and resources, making it difficult to disentangle the effectiveness of the online social networking component. Therefore, this study addressed the effectiveness of an 8-week beginners’ running program delivered entirely through a Facebook group in improving PA and cardiorespiratory fitness. Significant improvements were found in both groups at 2 months in MVPA; this increase was considerably larger in the intervention group (*P*=.04). Engagement with the Facebook group was relatively high compared with other online interventions. Further research is warranted to investigate the effectiveness of this delivery method in other health-related behaviors and with other population groups. The ease of use, low cost, and accessibility of a Facebook group make it a promising method for delivering socially supportive health and behavioral programs on a mass scale.

## Appendix

Multimedia Appendix 1 CONSORT‐EHEALTH checklist (V 1.6.1).

## Abbreviations

AAS: Active Australia Survey
BMI: body mass index
BPM: beats per minute
GLMM: generalized linear mixed model
MVPA: moderate to vigorous physical activity
PA: physical activity
RCT: randomized controlled trial
SCT: social cognitive theory
